# The Medical Profession and a Ministry of Health

**Published:** 1918-01-12

**Authors:** 


### The Hospital

The Workers' Newspaper of Administrative Medicine and Institutional Life, Administration, National Insurance and Health.

No. 1649, Vol. LXIII. SATURDAY, JANUARY 12, 1918 PRICE ONE PENNY

### THE MEDICAL PROFESSION AND A MINISTRY OF HEALTH.

The proposal to establish a Ministry of Health, though blessed with good will from many quarters, does not show much evidence of practical advance. Like many other promising schemes it suffers eclipse by the shadow of the national anxieties arising from the war. Even the most determined and pressing of its supporters have had in the meanwhile to abandon it, and the power which was resolved to bring it into being has been deflected, however unwillingly, from the provision of health to the supply of food. There are, however, still some sanguine souls who believe that the issue may be pushed to a complete and Parliamentary solution in spite of the immediate preoccupations of the hour. On the other hand are those who dwell on the complications of the question, and who point to the inability in existing circumstances of gaining anything like a full study of the subject by the medical profession. The opinions and proposals of the doctors are here of first importance, for no scheme can be expected to work effectively which has not their support and good will. Most of the schemes which have been advanced contemplate very close relations between the public authorities to be organised and created and the work of the general practitioner. Hence the question at once goes beyond the views and interests of the existing medical health authorities and concerns practically the main body of the profession. It may be quite true that the suggestion of a Ministry of Health has for years received the support of certain medical organisations which claim a more or less representative character. But until recently the profession generally has treated the proposal with benevolent good will rather than with close study. The reason for this attitude is an obvious one. Formerly the question was regarded as one which concerned mainly a limited number of doctors—namely, those engaged in the existing Public Health Services. But recent events have shown that the scope of the enterprise will suffer no such limitation. If efficient health organisation is to be brought into the lives and homes of the people the family practitioner must be enlisted in its service, and he must therefore have a voice in the definition of the conditions on which his co-operation can be secured. Thus, apart from the difficulties which arise from the circumstances of the day and hour, there is the special difficulty of obtaining the views and opinions of the medical profession, at least as this is represented by a wide suffrage. Many of the doctors are scattered to the four corners of the earth, and those who remain are pressed with duties which absorb all their time and attention.

A striking proof of the determination of the general practitioners to make their voices heard on this question was afforded at the last Representative Meeting of the British Medical Association. The Council of the Association had prepared a detailed scheme which they proposed to press on the attention of the Government. But this scheme had not been submitted to the Divisions of the Association and could not, therefore, claim to be the considered opinion of the members. Indeed, notice had been given of numerous amendments to it, and it was only under the strong plea of urgency that the Council obtained authority to proceed with the matter. Even then the approval was only a "general" one, and the Council was instructed to consider the alternative suggestions contained in the amendments which had been placed on the paper. At this time everyone believed that urgency undoubtedly existed. What seemed like an official pledge had been given, and it was confidently reported that a draft Bill was actually in existence. These were the circumstances in which the Representative Meeting allowed the details to pass out of their hands into the hands of the Council, and this position will undoubtedly have its consequences. For the event has shown that the need of urgency was not well founded, or that, if well founded at the time, new conditions have destroyed its value. Thus it may be accepted as practically certain that the members of the Association will claim the right which they consented to yield under a pressure that no longer exists. Official persons and organisations may arrange and organise such plans and schemes as seem to them good, but unless they carry with them the public opinion of the profession their ambitions may well in practice come to nought. At the present moment this public opinion cannot possibly be secured, and here is a sufficient reason why definite legislative action can hardly be a safe undertaking.

In this connection we note with interest that the Royal College of Physicians of Edinburgh has come to the conclusion that the scheme for the establishment of a Ministry of Health cannot be proceeded with in existing circumstances. The College can hardly claim to be a representative body, though it speaks with some confidence for a certain limited interest, and its judgment is not an unimportant one. The grounds for the conclusion reached by the College are probably not identical with those here stated. But the conclusion adds to a growing opinion that though the question is urgent a satisfactory answer to it cannot be hoped for until peace brings opportunities for full exploration and reflection.

